# Comprehensive Assessment of Eyes in Kidney Transplant Recipients after Recovering from COVID-19

**DOI:** 10.3390/life13102003

**Published:** 2023-09-30

**Authors:** Mateusz Ślizień, Paulina Sulecka, Leszek Tylicki, Zofia Janicka, Joanna Konopa, Zuzanna Ślizień, Alicja Dębska-Ślizień, Katarzyna Michalska-Małecka, Bogdan Biedunkiewicz

**Affiliations:** 1 Ophthalmology Clinic, Medical University of Gdańsk, 80-210 Gdańsk, Poland; mateusz.slizien@gumed.edu.pl (M.Ś.); popo@gumed.edu.pl (P.S.); zosi88@gumed.edu.pl (Z.J.); katarzyna.michalska-malecka@gumed.edu.pl (K.M.-M.); 2 Department of Nephrology, Transplantology and Internal Medicine, Medical University of Gdańsk, 80-952 Gdańsk, Poland; leszek.tylicki@gumed.edu.pl (L.T.); jkonopa@gumed.edu.pl (J.K.); zuzanna.slizien@gumed.edu.pl (Z.Ś.); adeb@gumed.edu.pl (A.D.-Ś.)

**Keywords:** kidney transplantation, COVID-19, optical coherence tomography, vessel density

## Abstract

Introduction: Patients after organ transplantation with COVID-19 have a higher risk of morbidity and mortality than patients in the general population. There are single studies that assess the eyes of COVID-19 patients, but there are no such studies on organ transplant recipients. The purpose of this study was to comprehensively examine the eyes of kidney transplant recipients (KTR) after recovery from mild to moderate SARS-CoV-2 infection. Methods: A total of 40 KTR after COVID-19 and 20 KTR without clinical and immunological symptoms of SARS-CoV-2 infection as a control group was qualified for the cross-sectional study. A total of 76 eyes from 38 KTR on an average of 7 weeks after COVID-19 and 36 eyes from 18 KTR from the control group were studied. The participants underwent an ophthalmological examination, and the retinal and choroid vessels and nerves were assessed by optical coherence tomography angiography. Results: We found a lower vessel density (VD) in the deep capillary plexus in the central part of the retina (VD deep central) of the study group. Women had significantly lower VD deep central in the study group (15.51 vs. 18.91, *p* < 0.001). Multivariate linear regression analysis confirmed an independent, negative impact of COVID-19 (*p* < 0.001) and female gender (*p* = 0.001) on VD deep central. Conclusion: The results of our study confirmed that changes in microcirculation induced by SARS-CoV-2 infection may affect the retinal vessels in KTR. Mild to moderate COVID-19 in KTR resulted in a significant reduction in VD deep central of the retina, with these changes being more common in females.

## 1. Introduction

In December 2019, an outbreak of coronavirus disease 2019 (COVID-19) caused by severe acute respiratory syndrome coronavirus 2 (SARS-CoV-2) broke out in Wuhan, China and was declared a pandemic on March 11, 2020 by the World Health Organization (WHO) [1]. By August 25, 2023, according to the WHO, the total number of confirmed new cases of infection worldwide was more than 769 million, with more than 2000 deaths reported in the last 28 days and a total number of deaths of more than 6.9 million [2]. Patients after organ transplantation are at increased risk of morbidity and mortality in the course of SARS-CoV-2 infection [3]. SARS-CoV-2 causes an infection that can lead to acute respiratory distress syndrome, thrombocytopathy, and endotheliopathy. Endotheliopathy, recognized as an organ-specific immune complication of SARS-CoV-2 infection, may develop early after the onset of COVID-19 (<2 weeks apart) or manifest later in the course of the disease and is associated with significant morbidity [4]. SARS-CoV-2 binds to cells through S proteins, which can bind, among others, to the angiotensin-converting enzyme 2 (ACE2) receptor present in most organs. The RAAS (renin–angiotensin–aldosterone system) and its components (ACE and ACE2 receptors) have been detected in many structures of the human eye, e.g., neurons and several retinal components, including retinal vascular endothelial cells, Müller cells, ganglion cells, and photoreceptor cells. This local regulatory system, such as the one present in the vascular endothelium, may be responsible for short- and long-term regional changes related to SARS-CoV-2 infection, including eyes [5].

The ophthalmic manifestation may dominate the COVID-19 course or appear after the virus has been eradicated. Pooled data of meta-analyses of 16 studies reporting 2347 confirmed COVID-19 cases showed that 11.64% of COVID-19 patients had ocular surface manifestations. Ocular pain (31.2%), discharge (19.2%), redness (10.8%), and follicular conjunctivitis (7.7%) were the main features [6]. Conjunctivitis was the most common ophthalmic symptom, especially in children in the pandemic with the original SARS-CoV-2 strain. The incidence of eyelid, eye surface, and anterior segment symptoms in COVID-19 patients in different studies ranged from 0.81% to 34.5%. The reasons for such large discrepancies were the different stages of the disease and its different severities, along with the lack of uniformity in the way of testing and data collection [7,8]. Visual impairment in terms of reduced visual acuity and “visual loss” was also reported in a few reports, but as the meta-analysis of Ripa et al. shows, that is rather a rare finding [9]. There have also been quite rare case reports describing orbital inflammatory diseases, for example, rhino-orbito-cerebral-mucormycosis co-infection COVID-19 patients [10].

Descriptions of disease processes related to the course of COVID-19 in the posterior segment of the eye are less common and have been described in the form of case reports [11]. The most common symptoms of COVID-19 in the retina are microvascular changes, such as cotton wool spots and retinal microhemorrhages, and most of these patients retained visual acuity and pupillary reflexes [12,13]. Paracentral acute median maculopathy (PAMM) and acute macular neuroretinopathy (AMN) may occur during SARS-CoV-2 infection, although the relationship between these conditions and COVID-19 requires further research [14]. Several studies have been conducted to evaluate microvascular changes in the retina, choroid, nerve fibers of the eyes, and changes in initial eye examinations in COVID-19 patients [15,16,17,18]. Such studies in the population of patients after kidney transplantation undergoing immunosuppression have not yet been performed. Given that retinal abnormalities may affect the final outcome of the patient’s visual health as well as may be a mirror of changes in the microcirculation of the whole organism, we undertook the study to investigate ocular manifestations such as maculopathy, vasculopathy, or neuropathy in kidney transplant recipients (KTR) who have recovered from COVID-19.

## 2. Materials and Methods

### 2.1. Design and Participants 

We conducted a cross-sectional and controlled study in KTR with a history of mild to moderate symptomatic COVID-19 under the control of our institution. The study involved 40 KTR who were infected with SARS-CoV-2 between December 2021 and June 2022 (Delta and Omicron variants), recovered from the infection, and were clinically stable before and during the study. A diagnosis of COVID-19 was confirmed by an RT-PCR test from nasopharyngeal/oropharyngeal swabs. No ophthalmic symptoms were observed in the enrolled patients. Seven patients were hospitalized due to moderate symptoms of COVID-19 with a decrease in saturation. (They did not require admission to the ICU.) A total of 24 patients with mild-to-moderate COVID-19 received molnupiravir as the prophylaxis of progressing to severe disease. A total of 20 KTR without clinical and immunological symptoms confirming SARS-CoV-2 infection served as a control group. Exclusion criteria were: type 1 or 2 diabetes, myopia > 6 diopters, glaucoma, retinal detachment, retinal vascular disease, macular degeneration, central serous retinopathy, other retinopathy, eye surgery within 6 months of evaluation, and opacity of the cornea, lens, or vitreous body. All laboratory and ophthalmological examinations in the study group after COVID-19 were performed, on average, 7 (2–17) weeks after the diagnosis of the disease. Ethics approval for the study was obtained at the Medical University of Gdansk (NKBBN/2014/2021).

### 2.2. Ophthalmologic Procedures

We investigated central retinal thickness (CRT), foveal avascular zone (FAZ) area, vessel density (VD) in superficial (SCP) and deep capillary plexuses (DCP), changes in the retinal nerve fiber layer (RNFL), and the ganglion cell complex (GCC) in the foveal and parafoveal areas. Patients underwent an ophthalmologic examination, which included best-corrected visual acuity (BCVA), where Snellen charts were used; intraocular pressure (IOP) measured by Goldmann applanation tonometry (GAT), which is currently the most widely accepted method used to measure IOP and is considered the gold standard tonometer in clinics; and anterior segment examination by slit-lamp microscope examination and fundus examination. The assessment of the fundus structures in each case was performed after dilating the patient’s pupil. For this purpose, Tropicamidum drops at a concentration of 1% were used, instilling the preparation twice at ten-minute intervals, and then the eye fundus was assessed after a twenty-minute waiting period for the action of the drops. The optic disc, retina, macular area, and blood vessels were assessed.

A slit lamp with a VOLK 90D lens was used for the fundus examination.

Retinal imaging was performed using optical coherence tomography (OCT), and VD was assessed using OCT angiography (OCTA) using an RTX1 adaptive optics retinal camera (RTX1). All scans were made with DRI-OCT Triton Plus OCT Angio (Topcon Inc., Tokyo, Japan). OCT files with poor quality evaluated by the Topcon OCT built-in software (IMAGEnet 6, Topcon Healthcare) with a score of TopQ image quality < 40 were excluded. Retinal thickness was assessed in its central area. Retinal thickness scans in the macular area were performed with a 7-by-7 protocol (512/256), OCTA with 6-by-6 scans, and RNFL scans with a 6-by-6 protocol. FAZ was measured in the superficial and deep plexuses. The FAZ was determined manually and was performed twice by two independent researchers (MŚ, PS), including the central fovea, where no clear and demarcated vessels were visible on the OCTA. The FAZ was defined as an area within the choroid plexus plate devoid of hyperreflective signal or flow in the center of the fovea.

OCTA parameters evaluated VD, which was measured in 5 sectors each for the SCP and DCP, such as central field 1 mm and the 1 mm inner superior/nasal/inferior/temporal field, using the ETDRS (Early Treatment Diabetic Retinopathy Study) grid subfields to define the areas of interest. VD was defined as the percentage of area occupied by vessels within the study area. VD measurement was calculated by the integrated software. We used the predefined boundaries provided by the software IMAGEnet Version 1.32.18683 for the SCP and the DCP analyses; the SCP was comprised between the inner limiting membrane (ILM) and 15.6 µm above the junction between the inner plexiform layer and the inner nuclear layer (IPL–INL), while the DCP was comprised 54.6 µm below the IPL–INL junction (between 15.6 µm and 70.2 µm).

RNFL and GCC were analyzed using the ophthalmic data system IMAGEnet 6 version 1.32.18683.

### 2.3. Data Collections

The records of patients were collected from the hospital’s electronic database by trained medical staff. The final data were verified by the major investigators. The collected data included demographic information, comorbidities, medications, history of renal replacement therapy, laboratory assessment, and COVID-19 severity. The Charlson comorbidity index (CCI) was calculated by summing the assigned weights of all comorbid conditions presented by the patients, according to the original formula [19]. For the purpose of research analysis, hospitalized patients were treated as having a more severe course. All of the laboratory measurements were performed by standard techniques in the Central Clinical Laboratory, University Clinical Centre, Gdańsk.

### 2.4. Statistical Analysis

Patient data were expressed as numbers (percentages) for categorical variables. The median (interquartile range; IQR) for continuous variables was given. We used the Chi-square test for categorical variables. Continuous variables were checked for normal distribution using Shapiro–Wilk. The *t*-test for normal distribution and the Mann–Whitney test for non-normally distributed continuous variables were used. Multivariable linear regression was used to determine the independent factors associated with the VD. Predictive covariates including age, sex, CCI, COVID-19, and COVID-19 severity were included in the regression model. Any variables that were at the significance level *p* less than 0.05 in univariate analyses were put in these models. The threshold of statistical significance was *p* < 0.05. Cohen’s kappa statistic was used to check the interobserver agreement (FAZ). Statistical package STATISTICA 13.3 in the Polish version (STATSOFT, Kraków, Poland) was used for statistical analysis.

## 3. Results

The study was completed by 38 patients (18 men, 47.3%), aged 52.5 (45–55) years, 58 (20–138) months after kidney transplantation. The average time from the onset of COVID-19 to the eye examination was 7 (2–17) weeks. The study was completed by 18 patients in the control group (10 men, 55.5%), aged 42 (36–49) years. The analysis included 76 eyeballs of COVID-19 patients and 36 eyeballs of the control group. The two groups did not differ significantly in terms of sex, comorbidities, and medications taken (Table 1).

Laboratory tests in both groups included examination of kidney transplant function markers (creatinine, eGFR), blood count parameters, d-dimer, fibrinogen, and c-reactive protein. The two compared groups did not differ significantly in the laboratory parameters studied (Table 2). 

**Figure 1:**
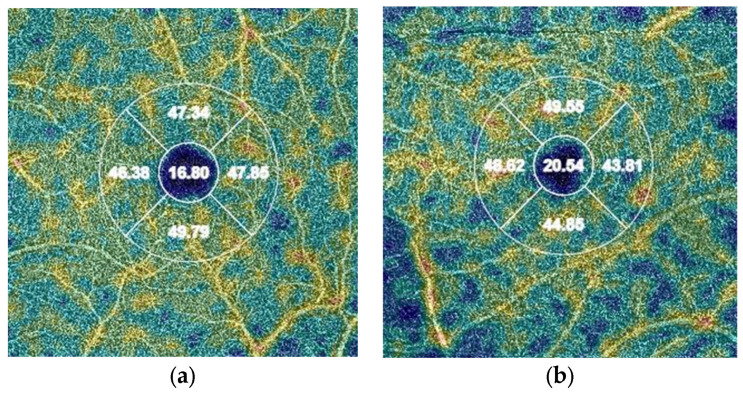
Differences in OCT measurements of deep vessel density in the central quadrant (values in %): (**a**) patient from the study group (COVID-19); (**b**) patient from the control group.

In the study group, a lower VD in the vessels was found (*p* = 0.015) in the DCP in the central part of the retina (VD deep central) (Figure 1). A downward trend was also observed for VD deep in the nasal, superior, and temporal quadrants (Table 3). We found no significant differences in VD in the SCP between groups. 

Women had significantly lower VD deep central values in the study group (15.51% vs. 18.91, *p* < 0.001). There was no association between age (r = 0.04; *p* = 0.72), comorbidity as measured by CCI (r = 0.14; *p* = 0.15), and severity of COVID-19 (*p* = 0.13) and VD deep central in univariate analyses. Multivariate linear regression analysis confirmed (*p* < 0.001) an independent, negative impact of COVID-19 disease (beta =−0.19; standard error beta = 0.094; *p* = 0.038) and female gender (beta = −0.32; standard error beta = 0.094, *p* = 0.001) on VD deep central.

There were no significant differences in IOP, RNFL, GCC, FAZ, CRT, slit-lamp testing, and BCVA (patients had a distance and near vision of 6/6 Snellen charts (0.0)) between groups. The high agreement between observers of 97.1% cases with Cohen’s kappa coefficient of 0.87 was achieved in the FAZ analysis (Table 4).

## 4. Discussion

Patients with chronic kidney disease have an increased risk of morbidity and mortality from COVID-19, compared to the entire population [20]. Moreover, persistent symptoms of infection in the form of post-COVID-19 syndrome may persist in these patients for many months after recovery [21]. Additionally, we demonstrated previously a significant deterioration in health-related quality of life in KTR who recovered from COVID-19, and the highest increase in health problems was reported for the “usual activity” and “pain/discomfort” dimensions [22]. The recent meta-analysis shows that a large percentage of patients from the general population report persistent eye symptoms in the form of “inability to focus vision” or “blurring/loss of vision” even at 6 months after recovery [23].

Ocular disorders are frequent among KTR and may affect 80% of patients. The main manifestations of ophthalmic complications are refractive errors, cataract formation, and sclerotic or hypertensive retinopathy. Besides immunosuppression and postoperative infection, aging is a high-risk factor in such cases. Ophthalmic abnormalities are mostly secondary to the patients’ primary kidney diseases, immunosuppressive therapies, metabolic disorders, cytomegaloviruses, herpes viruses, or other post-transplant infections [24]. As already mentioned, COVID-19 infection can exacerbate these problems and worsen the patients’ visual health.

The mechanism of entry and the action of the SARS-CoV-2 virus cause the respiratory system to be primarily involved. It has also been reported that the virus exhibits endothelial activity and neurotropism and therefore can infect and damage several systems and organs, including retinal tissues and vessels [13]. SARS-CoV-2 binds to ACE2 receptors and, upon entering host cells, induces acute respiratory distress syndrome (ARDS), induces a cytokine storm, and damages and worsens endothelial cell dysfunction. Hypoxia and inflammation lead to endothelial dysfunction and abnormal coagulation in small and large vessels [25,26,27]. Oxidative stress releases the vWF (von Willebrand factor) and causes hypoxic vasoconstriction, and direct cellular activation by viral transduction can lead to an increase in blood prothrombotic factors [28]. As a result of these processes, coagulopathy may develop, leading to vascular damage in organs, including the choroid and retina, which are rich in ACE and ACE2 receptors. SARS-CoV-2 infection may also be a trigger for anti-neutrophil cytoplasmic antibodies (ANCA)-associated vasculitis, both de novo and exacerbation, which is known to have an ophthalmological manifestation. The mechanisms that may trigger autoimmunity following SARS-CoV-2 infection include bystander killing, molecular mimicry, viral persistence, epitope spreading, and formation of neutrophil extracellular traps [29]. All of these mechanisms may consequently contribute to the damage to the structures of the eye.

In the present cross-sectional study, including KTR who recovered from COVID-19, we demonstrated significantly reduced VD in the DCP in the central part of the retina. Importantly, these changes were observed in patients with a relatively mild course of COVID-19, who did not present any ocular symptoms during the symptomatic period. A downward trend was also observed for VD deep in the nasal, superior, and temporal quadrants. Several studies have shown a reduction in VD of the retina among patients from the general population who recovered from COVID-19 [15,16,17,18]. These abnormalities were observed both in the SCP and the DCP. Unlike these studies, we also did not find a reduction in VD in the SCP. A potential explanation for our findings is that the inner layers of the retina have the highest sensitivity to hypoxic stress, compared to the outer layers, which are more resistant to hypoxia. The lack of changes in the SCP may have resulted from the mild course of COVID-19 and the short period from the onset of the disease [18,30,31]. The mild course of COVID-19 and, consequently, only slight, small, retinal vessel damage is also, in our opinion, the reason for the lack of changes in the FAZ area, RNFL, and CRT observed in our patients. As some studies showed, patients with more severe COVID-19 may experience not only vascular damage but also retinal neurodegenerative alterations that could be due to ischemia [32,33]. The mild course of the disease may also explain the lack of differences in VD between hospitalized and outpatient patients. Other studies reported that the severity of COVID-19 disease is associated with reduced retinal VD. Unlike other studies [31,34,35], we found no effect of age on the obtained VD values, which may be due to the relatively young age of the study population. The strength of our study is that we paid attention to the potential confounders which may affect VD. Patients with diabetes and eye diseases, which may have affected vascular damage or imaging quality, were not recruited. The study involved patients with arterial hypertension, which is present in most KTR; however, the incidence was the same in both groups and did not affect the results of the study. Therefore, any effects on retinal VD could be more likely attributable to COVID-19 infection.

Our study showed an independent effect of gender on retinal vascular damage. Women had significantly lower VD deep central values as compared to men. On the one hand, this finding is surprising when we take into account that men are exposed to a more severe course of COVID-19 [36]. On the other hand, other authors noticed a similar relationship as we did [15,37]. For instance, Kal et al. reported that women 6 months after recovery from COVID-19 presented both decreased vascular density in superficial and deep layers of the retina, as well as an increased FAZ area, compared to men [15]. The explanation is not obvious. Some authors point to slower recovery and regeneration processes in women. This may be indicated by the longer persistence of neurological and mental COVID-19 symptoms in women observed in some studies, such as fatigue, musculoskeletal pain, “brain fog”, and alopecia in the form of post-COVID-19 syndrome [33,38]. Both immunological and hormonal factors may be responsible for this. Estrogen and progesterone are known regulators of blood flow through the retina and choroid and play key roles in regulating vascular tone through endothelin-1 and NO production [39]. Sex hormones may also play a role in perpetuating the hyperinflammatory status of the acute phase even after recovery [30]. A stronger production of IgG antibodies in females in the early phase of COVID-19 may play a role in better prognosis in the acute phase of the disease, but it also contributes to the pathogenesis of persistent abnormalities and symptoms [37,38,40]. In the light of the above results, further studies assessing blood circulation in the retina and choroid based on gender differences are needed.

The significance of our study stems from the fact that, for the first time, the impact of a COVID-19 illness on damage to the small vessels of the retina was demonstrated in the KTR population. Moreover, we showed that microvascular abnormalities can occur, even with mild disease, in patients without any ocular symptomatology. Our study showed that OCTA imaging may be useful in detecting these changes in KTR. The retina, which is one of the most perfused organs, and its vasculature is resistant to autonomic regulation and is an ideal place to assess changes in microcirculation. The clinical relevance of our results may also extend beyond the ocular complications of COVID-19, and the abnormalities found in the retina may reflect microvascular changes in other organs. In particular, this may apply to tissues that are embryologically and structurally similar to the retina, such as the brain, where a significant homology on the anatomy and regulatory processes of the micro-vasculature is observed [41]. As we know, changes in microcirculation could also be occurring in the brain, and neurological symptoms may occur both in the acute phase of COVID-19 and after virus eradication, e.g., in the form of “brain fog” in the post-COVID-19 syndrome [42]. Future studies should evaluate the role of OCT and in identifying such abnormalities.

There are some limitations in our study. The small number of patients in both groups may have limited the power to detect small differences between variables. The cross-sectional nature of the study prevents the ability to monitor the evolution of the retinal alterations found over time. Additionally, our results may not have found any associations, as our cohort included only a middle-aged population with little comorbidities and mild-to-moderate courses of COVID-19.

## 5. Conclusions

The results of our study confirmed that changes in microcirculation induced by SARS-CoV-2 infection may affect the retinal vessels in KTR. We found significantly reduced VD in the DCP in the central part of the retina in patients with a history of mild-to-moderate COVID-19. These changes were significantly more common in women. Careful retina examinations using OCT and OCTA of patients who recovered from COVID-19 should be considered to assess the effects of COVID-19 on microcirculation. As time passes, other long-term consequences of COVID-19 on eyes in KTR will likely be revealed.

## Figures and Tables

**Table 1:** Characteristics of study patients.

Parameter	Study Group (n = 38)	Control Group (n = 18)	*p*
Sex	18 m (47.3%)	10 m (55.5%)	0.74
Age	52.5 (45–55)	42 (36–49)	0.003
RRT (months)	234 (221–252)	229.5 (215.5–259.5)	0.89
KTx vintage (months)	58 (20–138)	66 (19–146)	0.84
Charlson comorbidity index (CCI)	3 (2–3)	2 (2–2)	0.35
Arterial hypertension	37	16	0.75
Number of hypotensive drugs	2	1.5	0.97
Stroke	1	0	0.95
Coronary artery disease	3	1	0.67
Peripheral artery occlusive disease	0	0	0.59
Statin therapy	9	7	0.22
RAAS therapy	9	5	0.72
ASA	7	6	0.18
LMWH	1	0	0.45
NOAC	1	0	0.45

Parameters expressed as median (Me) and interquartile range (IQR). ASA—acetylsalicylic acid; KTx—kidney transplantation; n—number of patients; NOAC—new oral anticoagulants; RAAS—renin–angiotensin–aldosterone system; RRT—renal replacement therapy; LMWH—low molecular weight heparin.

**Table 2:** The laboratory tests.

Parameter	Study Group (n = 38)	Control Group (n = 18)	*p*
Creatinine (mg/dL)	1.34 (0.97–1.67)	1.39 (0.99–1.79)	0.33
eGFR (ml/min/1.73 m^2^) CKD-EPI	52.5 (44–68)	71 (45–76)	0.62
CRP (mg/L)	1.73 (0.86–2.53)	1.04 (0.48–1.82)	0.43
D-dimer (ng/mL)	394 (243–783)	509 (348–874)	0.71
Fibrinogen (g/L)	3.02 (2.8–3.49)	2.98 (2.54–3.35)	0.73
Hemoglobin (g/dL)	13.25 (12.5–14.8)	14.06 (12.8–15.3)	0.11
Hematocrit (%)	40.35 (37.9–43.6)	43.5 (39.8–45.9)	0.49
Platelets (10^9^/L)	219.5 (199–274)	233 (180–276)	0.37
WBC (10^9^/L)	8.0 (6.93–9.37)	8.8 (16.64–10.23)	0.54
Lymphocytes (10^9^/L)	2.3 (1.53–2.62)	1.91 (1.27–2.41)	0.48

Parameters expressed as median (Me) and interquartile range (IQR). CRP—c-reactive protein; eGFR—estimated glomerular filtration rate; n—number of patients; WBC—white blood cells.

**Table 3:** The vessel densities in the deep and superficial capillary plexuses of the retina.

Parameter	Study Group (n = 76)	Control Group (n = 36)	*p*
DCP central (%)	16.31 (6.37–29.58)	20.31 (9.89–29.35)	0.015 *
DCP superior (%)	48.28 (33.87–54.74)	49.19 (43.16–52.21)	0.08
DCP inferior (%)	47.05 (48.06–35.38)	48.2 (39.91–53.53)	0.49
DCP nasal (%)	45.86 (47.04–32.6)	47.92 (40.15–51.14)	0.06
DCP temporal (%)	45.78 (46.04–31.53)	47.73 (42.27–51.22)	0.07
SCP central (%)	21.34 (13.39–30.61)	21.45 (13.89–30.4)	0.87
SCP superior (%)	46.70 (35.03–51.3)	48.08 (36.88–51.22)	0.57
SCP inferior (%)	46.31 (35.67–53.28)	47.4 (36.9–49.79)	0.79
SCP nasal (%)	45.84 (31.22–50.98)	47.08 (32.64–52.4)	0.38
SCP temporal (%)	47.69 (34.81–52.66)	48.25 (36.73–55.1)	0.48

n—Number of patients; *—statistically significant *p* < 0.05; vessel density (VD) in superficial (SCP) and deep capillary plexuses (DCP).

**Table 4:** The foveal avascular zone, central retinal thickness, retinal nerve fiber layer, and ganglion cell complex.

Parameter	Study Group (n = 76)	Control Group (n = 36)	*p*
FAZ superficial (µm^2^)	285.50 (114–602)	275 (60–510)	0.51
FAZ deep (µm^2^)	402 (140–770)	397.5 (185–660)	0.35
CRT (µm)	234 (182–285)	229.5 (175–278)	0.59
RNFL (µm)	100 (64–128)	99.5 (41–120)	0.47
GCC (µm)	101.5 (63–124)	103.5 (70–135)	0.33

n—Number of patients; foveal avascular zone (FAZ) area; central retinal thickness (CRT); retinal nerve fiber layer (RNFL); ganglion cell complex (GCC).

## Data Availability

Data are available upon reasonable request to the corresponding author.
